# Potential local adaptation of corals at acidified and warmed Nikko Bay, Palau

**DOI:** 10.1038/s41598-021-90614-8

**Published:** 2021-05-27

**Authors:** Haruko Kurihara, Atsushi Watanabe, Asami Tsugi, Izumi Mimura, Chuki Hongo, Takashi Kawai, James Davis Reimer, Katsunori Kimoto, Marine Gouezo, Yimnang Golbuu

**Affiliations:** 1 Department of Chemistry, Biology, and Marine Science, Faculty of Science, University of the Ryukyus, 1 Senbaru, Nishihara, Okinawa 903-0213 Japan; 2 Department of Transdisciplinary Science and Engineering, School of Environment and Society, Tokyo Institute of Technology, 2-12-1 W8-13, Meguro, Tokyo 152-8550 Japan; 3 The Ocean Policy Research Institute, The Sasakawa Peace Foundation, 1-15-16 Toranomon, Minato, Tokyo 105-8524 Japan; 4 Research Institute for Global Change, Japan Agency for Marine-Earth Science and Technology (JAMSTEC), 2-15, Natsushima-cho, Yokosuka, Kanagawa 237-0061 Japan; 5 Palau International Coral Reef Center, 1 M-Dock Road, PO Box 7086, Koror, 96940 Republic of Palau

**Keywords:** Marine biology, Tropical ecology

## Abstract

Ocean warming and acidification caused by increases of atmospheric carbon dioxide are now thought to be major threats to coral reefs on a global scale. Here we evaluated the environmental conditions and benthic community structures in semi-closed Nikko Bay at the inner reef area in Palau, which has high *p*CO_2_ and seawater temperature conditions with high zooxanthellate coral coverage. Nikko Bay is a highly sheltered system with organisms showing low connectivity with surrounding environments, making this bay a unique site for evaluating adaptation and acclimatization responses of organisms to warmed and acidified environments. Seawater *p*CO_2_/Ω_arag_ showed strong gradation ranging from 380 to 982 µatm (Ω_arag_: 1.79–3.66), and benthic coverage, including soft corals and turf algae, changed along with Ω_arag_ while hard coral coverage did not change. In contrast to previous studies, net calcification was maintained in Nikko Bay even under very low mean Ω_arag_ (2.44). Reciprocal transplantation of the dominant coral *Porites cylindrica* showed that the calcification rate of corals from Nikko Bay did not change when transplanted to a reference site, while calcification of reference site corals decreased when transplanted to Nikko Bay. Corals transplanted out of their origin sites also showed the highest interactive respiration (R) and lower gross photosynthesis (Pg) to respiration (Pg:R), indicating higher energy acquirement of corals at their origin site. The results of this study give important insights about the potential local acclimatization and adaptation capacity of corals to different environmental conditions including *p*CO_2_ and temperature.

## Introduction

Increases in atmospheric carbon dioxide (CO_2_) simultaneously cause both ocean warming and acidification, which are now thought to be the major threats to coral reefs on a global scale^1,2^. Coral mass bleaching events are now occurring more frequently compared to the past, and ocean warming is predicted to further increase the susceptibility of corals to bleaching in the coming decades^3,4^. Some studies have indicated that zooxanthellate corals may have the ability to adapt to high temperature environments, such as by shuffling to Symbiodiniaceae types that have higher tolerances to warmer temperature^5–7^. In addition to global warming, increases of seawater partial pressure of CO_2_ (*p*CO_2_) and decreases of aragonite calcium carbonate saturation (Ω_arag_) have been reported to decrease calcification rates of most reef calcifiers including corals and crustose coralline algae (CCA)^8,9^. Meanwhile, it has been shown that the tolerance of organisms to high *p*CO_2_ can differ among species and even within species^10^. Hence, there is now wide interest in understanding how reef organisms will respond to ocean warming and acidification at the community level, and to examine if organisms are able to acclimatize or adapt to these environmental changes.

**Figure 1:**
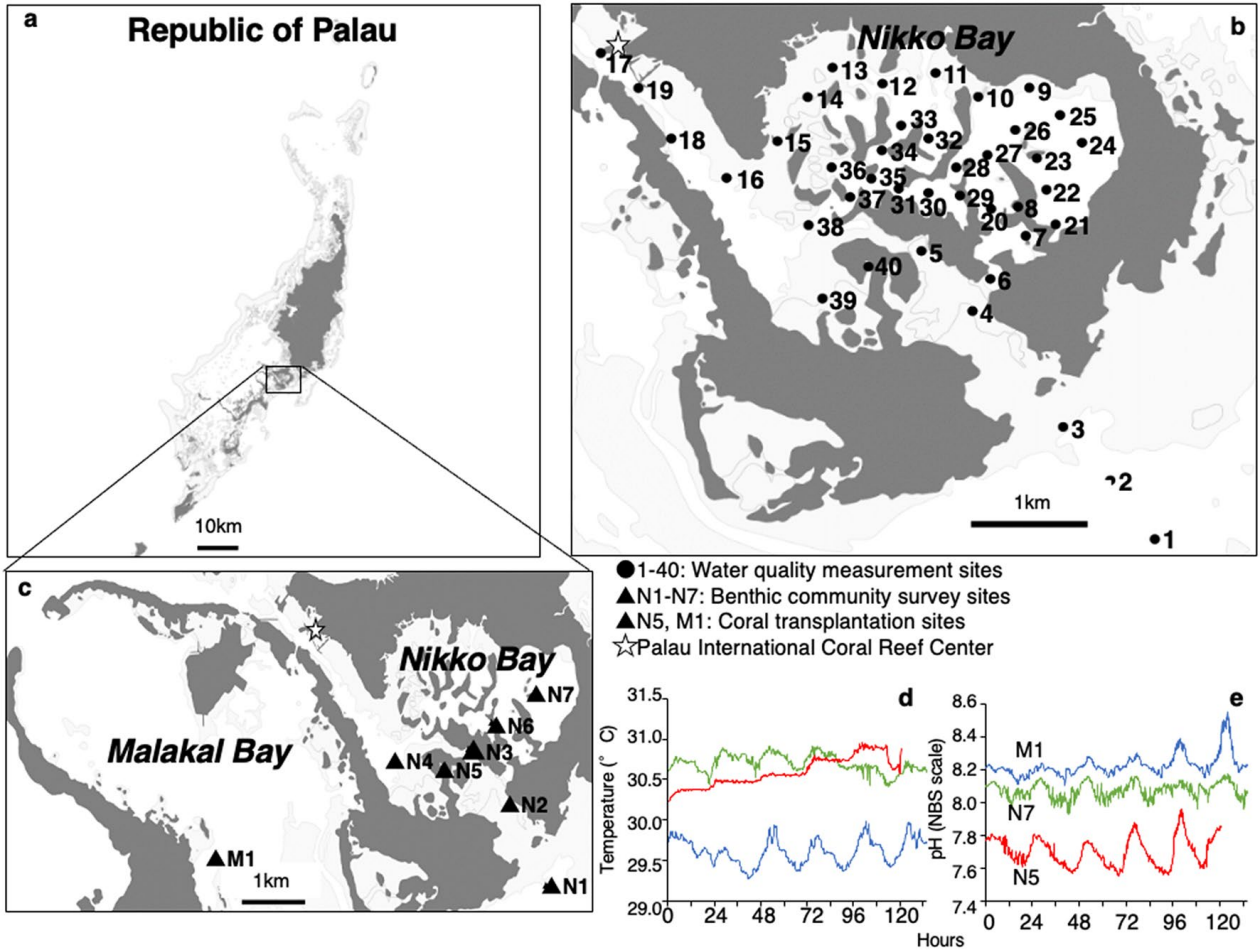
Map showing study sites and seawater temperature and pH at 3 sites (M1, N7, N5). (**a**) Map of the Republic of Palau. (**b**) The 40 locations where seawater quality was measured around Nikko Bay. (**c**) The seven sites (N1-N7) where benthic communities were surveyed and the reference site at Malakal Bay (M1) where the coral *Porites cylindrica* experiment was conducted. The coral *P. cylindrica* was sampled from sites M1 and N5 for reciprocal transplantation experiment. (**d**) Diurnal seawater temperature and (**e**) pH (total scale) measured at Malakal Bay (M1) and two sites at Nikko Bay (N7 and N5). The figure was created using QGIS 3.8.1 (https://www.qgis.org).

Here we investigated a semi-closed bay (Nikko Bay) in the inner reef area in Palau (Fig. 1) with high *p*CO_2_ and high-temperature conditions, and counter-intuitively, high coral coverage^11,12^. The elevated *p*CO_2_ observed in this bay has been suggested to be mainly due to natural biological activity and seawater circulation^12,13^. Nikko Bay’s corals have shown little evidence of bleaching during the 1998 mass bleaching on other reefs of Palau and during the 2010 thermal stress event^11,14^. A global mass bleaching event occurred in 2016–2017^3^, however, it only affected eastern parts of Micronesia^15^. Palau’s SST rapidly shifted in March 2016 and reached above 30 °C in June 2016 but quickly decreased afterward and bleached corals quickly recovered instead of dying^16^, leading to no decrease in percentage of live coral cover at the scale of the archipelago^17^.

CO_2_ vent sites^18–20^ and naturally acidified sites^21–23^ have been found on coral reefs and utilized as essential models for evaluating the effects of ocean acidification at the ecosystem level. However, most of these systems are open or semi-closed lagoon ecosystems with significantly shorter residence times compared with that of Nikko Bay, and thus they may continuously receive recruitment from the surrounding ocean; this may limit local adaptation of organisms to high *p*CO_2_ conditions. On the other hand, Nikko Bay is of particular interest because it is a highly sheltered system with a long seawater residence time of 71 days, and with organisms showing low connectivity with surrounding populations^12,24^. Additionally, the seawater conditions found in this bay have been suggested to have been maintained at least for the past 150–500 years^12^ and hence, long-term selection of coral larvae may have allowed local adaptation or acclimatization responses to environmental conditions found within this bay. Finally, this unique bay provides opportunities to evaluate the effects of the co-stressors of ocean warming, ocean acidification, low oxygen and high chlorophyll-*a* (Chl-*a*) levels, at the community scale. Recent studies in mangrove habitats^22,25^, tidal inshore reefs^26^, and upwelling systems^27^ have demonstrated the need and importance of evaluating these multiple co-varying factors in understanding the mechanisms sustaining complex ecosystems and in predicting future coral reef scenarios^28^.

Here we evaluated the environmental conditions and benthic community structures along with the Ω_arag_ gradient found within the bay. We also conducted reciprocal transplantation experiments of the most dominant coral species, *Porites cylindrica*, to evaluate the potential acclimatization and adaptive responses of corals to warm and acidified conditions.

## Results and discussion

**Figure 2:**
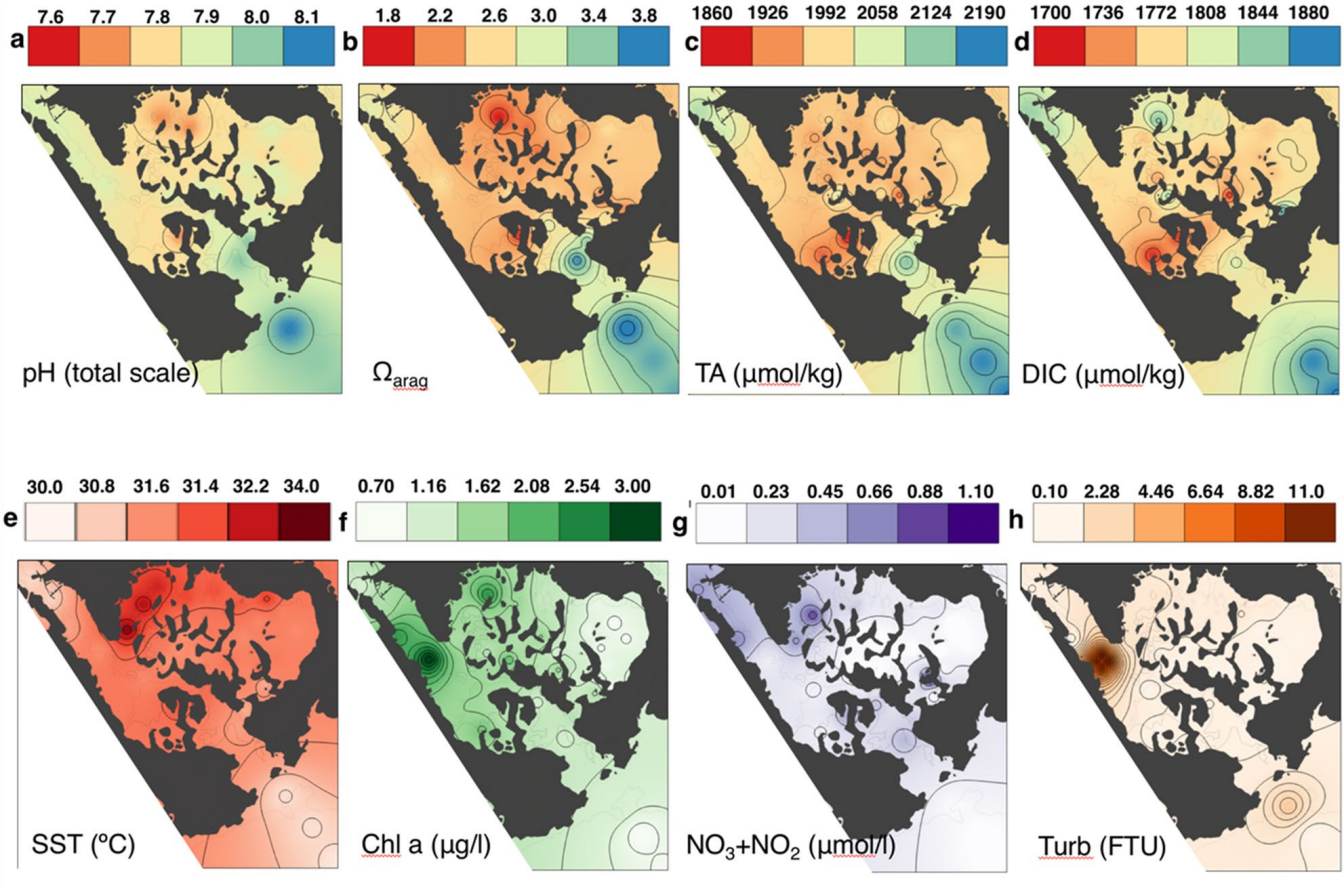
Spatial gradient of (**a**) pH (total scale), (**b**) aragonite saturation state (Ω_arag_), (**c**) total alkalinity (TA, μmol equivalent kg^−1^), (**d)** dissolved inorganic carbon (DIC, μmol kg^−1^), (**e**) sea surface temperature (SST, °C), (**f**) chlorophyll-*a* (Chl-*a*, µg L^−1^), (**g**) nitrate + nitrite (NO_2_^−^  + NO_3_^−^, μmol L^−1^) and (**e**) turbidity (FTU) in sea surface water during daytime around Nikko Bay. See Tables S1 and S2 for details. The figure is created using QGIS 3.8.1 (https://www.qgis.org).

Seawater surface pH (total scale), Ω_arag_ and temperatures (SST) showed a strong gradient at the entrance into the bay (Fig. 2a, b, e) and the seawater pH range (7.65–8.02) observed within the bay was equivalent to the ocean pH value from present to the value expected by the end of this century (IPCC 2013, RCP 8.5)^29^. The mean daytime seawater temperature within the bay was significantly warmer (31.8 ± 0.6 °C, mean ± S.D.) and had lower pH (7.83 ± 0.06), lower Ω_arag,_ (2.44 ± 0.34) and higher *p*CO_2_ (619 ± 104 μatm) compared to parameters outside the bay (30.4 ± 0.1 °C, 8.02 ± 0.02, 391 ± 31 μatm, 3.63 ± 0.14, Wilcoxon-test, *p* < 0.01, Tables S2), respectively. The seawater pH at Nikko Bay showed diurnal variation, ranging from 0.05 to 0.25, which was consistent with the range observed outside the bay (Fig. 1e, Table S2) and at other coral reefs^30^. This contradicts with most conditions at CO_2_ vents where the seawater pH is highly variable temporally^18–20^. Average Chl-*a* and nutrient concentration values inside Nikko Bay were slightly but significantly higher than those outside the bay (Wilcoxon-test, *p* < 0.01, Fig. 2f–g, Table S1).

**Figure 3:**
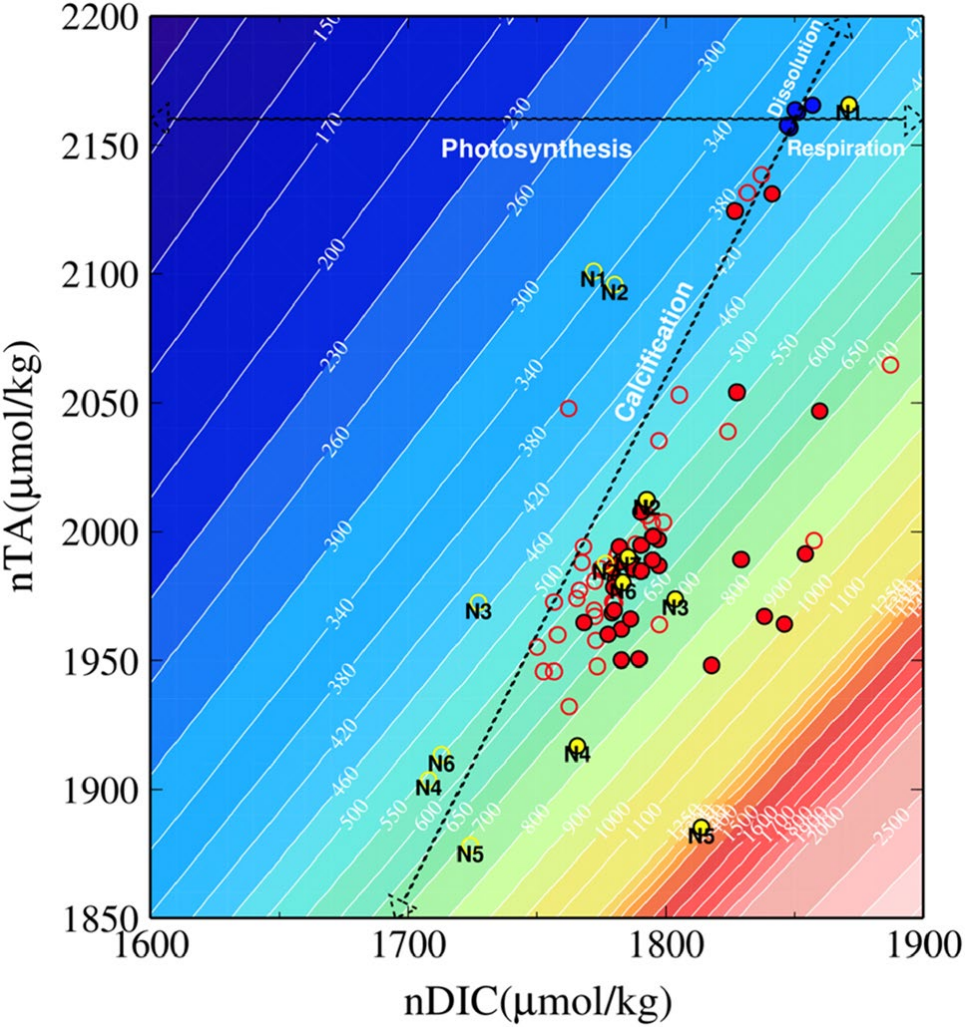
Salinity normalized TA-DIC diagram for the seawater collected around Nikko Bay. Data are normalized at the mean Nikko Bay salinity of 33.02 during the survey. Yellow symbols indicate data collected from N1 to N7 (Fig. 1c) during daytime (open) and at nighttime (filled). Red symbols indicate samples collected at other sites in Nikko Bay during daytime (open) and at nighttime (filled). Blue symbols indicate data collected at far offshore sites as end members. Trend lines indicating calcification, dissolution, photosynthesis, and respiration are drawn from these offshore end member values. Contours indicate *p*CO_2_ isolines calculated at S = 33.02 and T = 30 °C.

Daytime average total alkalinity (TA) and dissolved inorganic carbon (DIC) were significantly lower within the bay compared to outside the bay (Wilcoxon-test, *p* < 0.01, Fig. 2c, d, Table S2) and the TA-DIC diagram indicated that the low pH and high *p*CO_2_ within Nikko Bay were mainly caused by low seawater TA due to calcification and by high DIC due to respiration (Fig. 3). By using the calculated mean water residence time within the bay (71 days^12^), mean net calcification (Gn) and net primary production (Pn) rates within the bay were calculated to be 22.7 mmol CaCO_3_ m^−2^ d^−1^ and − 6.9 mmol C m^−2^ d^−1^. These values were lower than the net calcification rates found at most reefs^31^, however the positive net calcification at seawater Ω_arag_ of 2.44 within the bay contradicts with previous studies suggesting that coral reef formation is restricted to seawater Ω_arag_ higher than ca. 2.8 (*p*CO_2_ lower than 560 μatm)^32^, and also with CO_2_ seep studies indicating that reef development ceases where pH is lower than 7.7 (Ω_arag_ 2.1) in Papua New Guinea^18^, and lower than 7.9 in the Mariana Islands^20^. When CO_2_ gas exchange is considered, the heterotrophy of Nikko Bay becomes even higher. Although there are no wind speed data measured directly inside Nikko Bay, wind speed measured at PICRC station was 0.51 ± 0.80 m s^−1^ (N = 4320) during this period, and wind can be expected to be weaker in Nikko Bay, which is surrounded by islands. Using the average *p*CO_2_ of seawater in the bay (670 μatm, Table S2), atmospheric pCO_2_ (390 μatm, calculated from mole fraction of CO_2_ in dry air data collected at Guam), and gas exchange coefficient^33^ utilized; gives the largest gas flux under this condition), the *p*CO_2_ flux was calculated to be about 5.6 mmol m^−2^ d^−1^. Using this value, the Pn in Nikko Bay would be approximately -12.5 mmol m^−2^ d^−1^.

**Figure 4:**
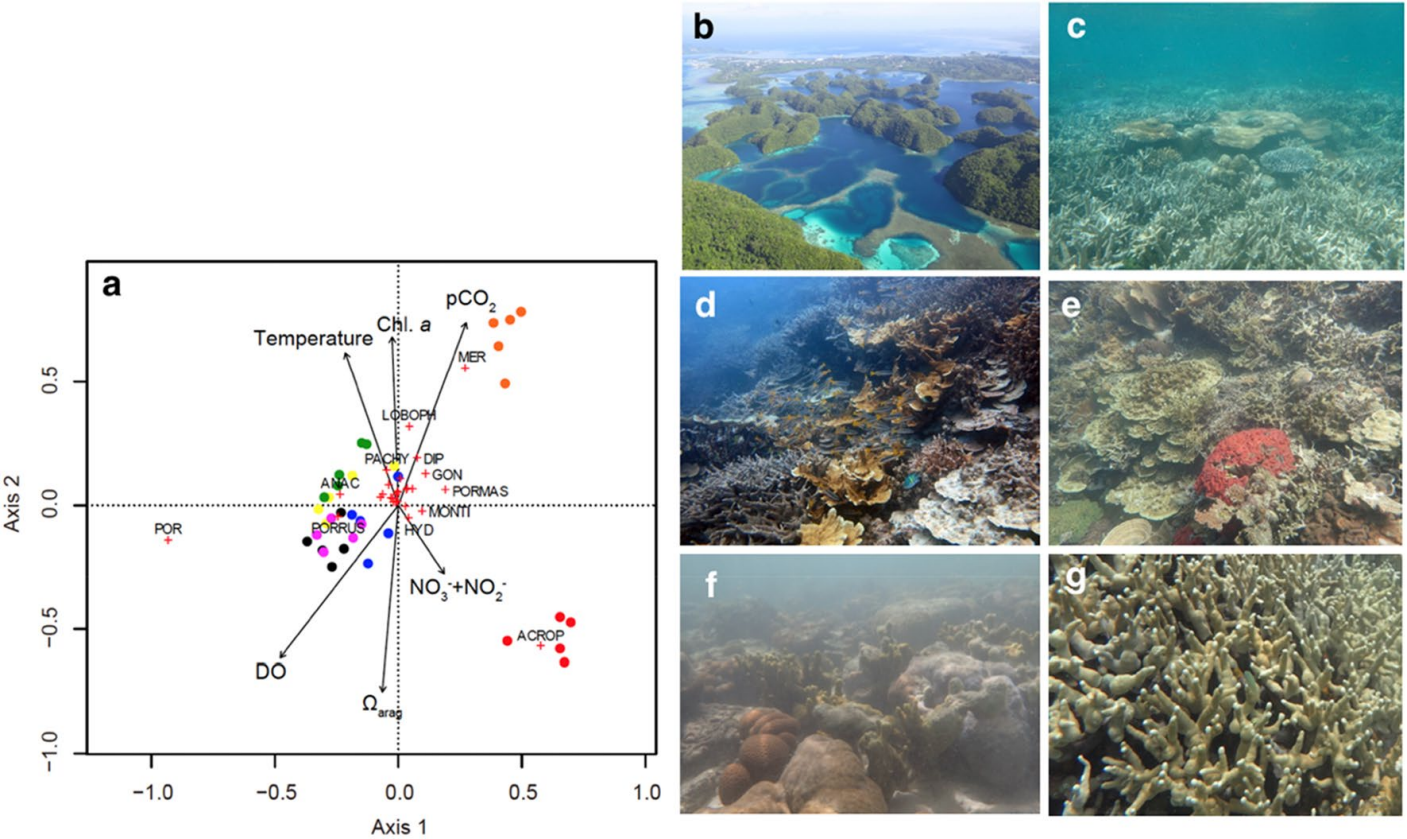
Redundancy analysis (RDA) for water quality and hard coral community at seven sites around Nikko Bay (N1-N7) and image of sites with different benthic communities. (**a**) Ordination of coral community based on redundancy analysis (Eigenvalue axis 1: 0.1795, Eigenvalue axis 2: 0.1035). Arrows represent significant seawater environmental variables, and their direction and length indicate their contributions to variation along those axes. Dots indicate transect lines with colors distinguishing study sites: red: N1, black: N2, blue: N3, yellow: N4, light blue: N5, green: N6, pink: N7. Genera/families of hard corals are indicated by plus symbols; selected genera are indicated by codes: LOBOPH: *Lobophyllia* spp., ACROP: *Acropora* spp., ANAC: *Anacropora* spp., MONTI: *Montipora* spp., MER: Merulinidae, DIP: *Dipsastrea* spp., GON: *Goniastrea* spp., HYD: *Hydnophora* spp., PACHY: *Pachyseris* spp., POR: branching *Porites* spp., PORMAS: massive *Porites* spp., PORRUS: *Porites rus*. (**b**) aerial image of Nikko Bay, (**c**) image of site N1 (Ω_arag_ = 3.51), a reef outside of Nikko Bay mainly covered by *Acropora* spp*.*, (**d**) image of site N6 (Ω_arag_ = 2.41) within Nikko Bay mainly covered by *Porites* spp*., Pachyseris* spp*. and Anacropora* spp*.*, (**e**) image of site N7 (Ω_arag_ = 2.36) (**f**) image of site N5 (Ω_arag_ = 1.28) mainly covered by Merulinidae and *Porites* spp, and (**g**) image of the most dominant coral *Porites cylindrica*.

To evaluate the correlation among seawater carbonate chemistry and benthic community structure, six sites (N2-N7) along with the Ω_arag_ gradient (1.28–3.51) inside the bay and one site outside of Nikko Bay (N1) were selected for benthic community observation (Fig. 1, Table S3). Even though the seawater inside the bay was warmer and more acidified than the seawater outside the bay, hard coral coverage inside the bay (N2-N7) ranged from 34 to 82%, while the coverage at the N1 site outside the bay was 24% (Tables S4). There was no significant correlation between seawater Ω_arag_ and scleractinian hard coral cover (GLM, *p* = 0.97), CCA (GLM, *p* = 0.68), macroalgae, or seagrass coverage (GLM, *p* = 0.06, Fig. S1, Table S5). On the other hand, there was a significant increase in soft coral (= octocoral) coverage with an observed decrease of Ω_arag_ (GLM, *p* = 0.01), which follows previous results at a CO_2_ vent at Iwotorishima in southern Japan showing high coverage of soft coral at a high *p*CO_2_ site^19^. Turf algae coverage increased with Ω_arag_ (GLM, *p* = 0.04), contradicting previous observations at a CO_2_ vent in the Mariana Islands^20^ (Table S5, Fig. S1). In Nikko Bay, the coral community was found to differentiate along with the Ω_arag_ gradient observed from the outer reef to the inner reef area^23^. Here we found that although coral coverage was not affected, the hard coral community structure showed differentiation among sites within the inner reef bay area, and this structure was mainly predicted by seawater Ω_arag_, dissolved oxygen (DO), Chl-*a,* nitrate plus nitrite (NO_3_^−^  + NO_2_^−^) concentration, *p*CO_2_ and temperature (Fig. 4, Table S6). Site N1 (outside of the bay) was characterized by high Ω_arag_ (3.51), low *p*CO_2_ (395 μatm), low temperature (29. 3 °C), low Chl-*a* (0.55 μg/L), high DO (6.09 mg/L), and was dominated by *Acropora* spp. (coverage 16.5 ± 4.1%), while site N5 was characterized by low Ω_arag_ (1.28), high *p*CO_2_ (1,305 μatm), high temperature (30.5 °C), high Chl-*a* (1.68 μg/L), low DO (4.52 mg/L), and was dominated by Merulinidae spp. (15.6 ± 5.2%, Fig. 4, Tables S3–S5). Both *Acropora* spp. and massive *Porites* showed a slight but positive correlation with seawater Ω_arag_ (GLM, *p* = 0.04, Fig. S1, Table S5), suggesting that species belonging to these genera are sensitive to OA, though other environmental factors may also have interactively affected the coverage of those species. Branching *Porites* (mainly consisting of *Porites cylindrica*) showed the highest coverage, accounting for 22 to 79% of hard coral cover inside the bay (Fig. 4g, Table S4), and there was no significant correlation with branching *Porites* spp*.* and Ω_arag_ (GLM, *p* = 0.16, Table S5. These results suggest that the high coral cover observed within Nikko Bay is related to the potential acclimatization or adaptation capacity of corals such as *P. cylindrica* to high *p*CO_2_ (low Ω_arag_) seawater.

**Figure 5:**
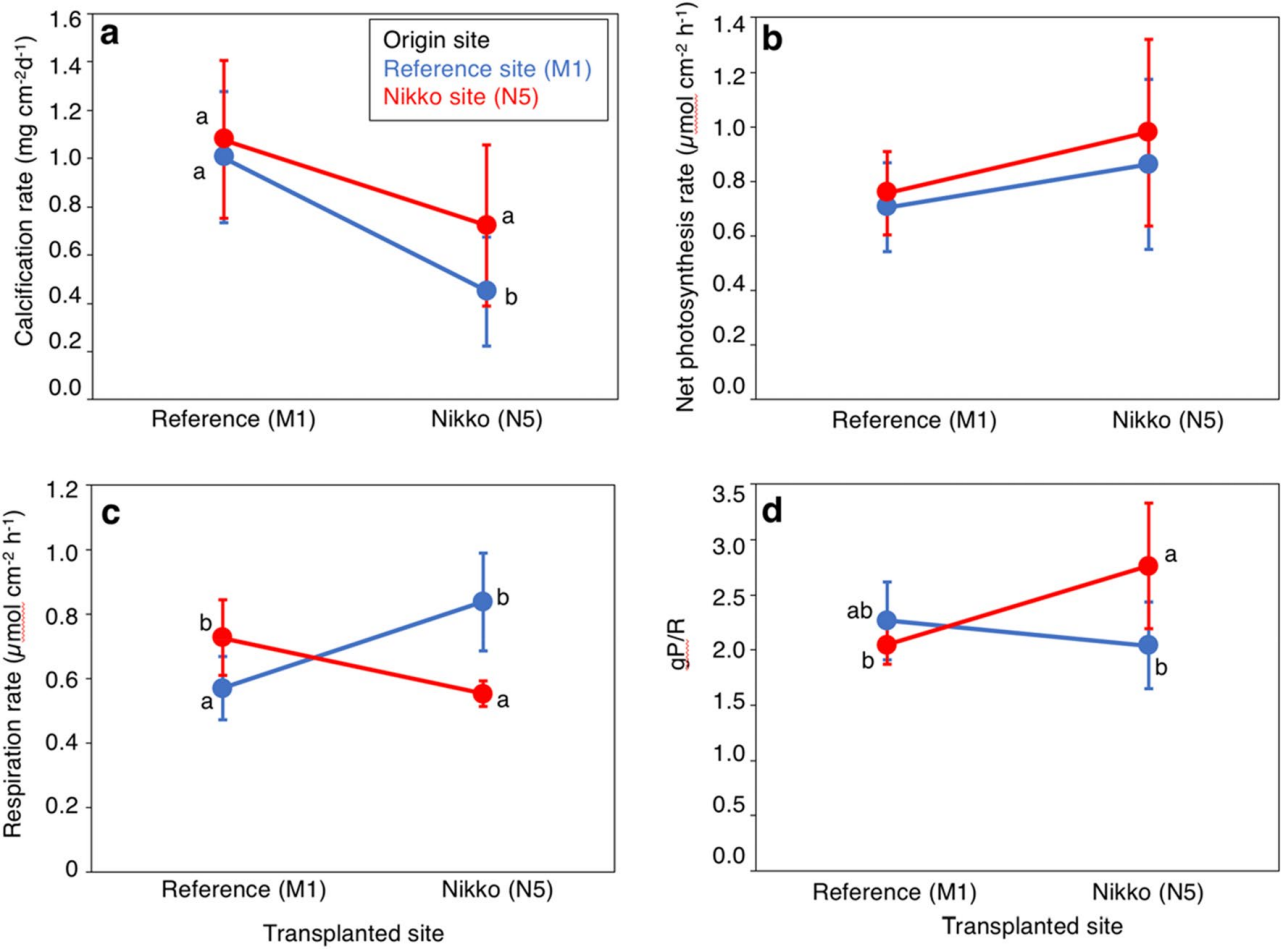
Metabolism of the coral *Porites cylindrica* reciprocally transplanted between the reference site (M1) and Nikko Bay site (N5). **(a**) Calcification rate (n = 12), (**b**) net photosynthesis rate (Pn, n = 9), (**c**) respiration rate (R, n = 9), and (**d**) gross photosynthesis ratio to respiration (Pg : R, n = 9) of *P. cylindrica* originated from the reference site M1 (blue) and Nikko Bay site N5 (red), and reciprocally transplanted for 18 days to either sites. Bars with different lower letters show significant differences among them (Tukey–Kramer HSD, *p* < 0.05).

**Figure 6:**
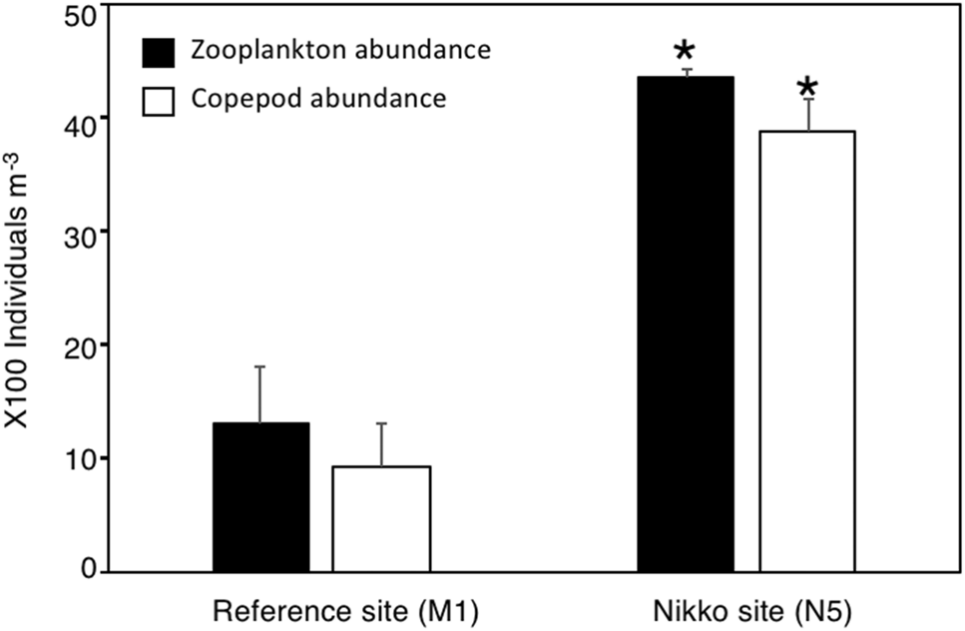
Zooplankton (black bar) and copepod (write bar) abundances at reference site (M1) and Nikko Bay site (N5). Average and S.D. for 3 nights plankton net sampling at each site. Asterisks show significant differences between the two sites (student *t*-test, *p* < 0.05).

To determine this possibility, colonies of *P. cylindrica* were reciprocally transplanted between two inner reef bays; a reference site at Malakal Bay (site M1) and a site in Nikko Bay (site N5, Fig. 1) that had different seawater temperatures and *p*CO_2_ conditions (Table S7). As a result, it was found that while the calcification rate of *P. cylindrica* originating from M1 significantly decreased when transplanted to N5, the calcification rate of corals from N5 did not show significant differences when transplanted to either M1 and N5 (Fig. 5a, Table S8). Most previous tank experiments have reported a decrease of calcification rates of *P. cylindrica* at high *p*CO_2_^34,35^ or under high *p*CO_2_ and high temperature conditions^36^. Additionally, in contrast to massive *Porites*, *P. cylindrica* was found to have less capacity of up-regulating calicoblastic calcifying fluid pH, suggesting a high sensitivity to increases of seawater *p*CO_2_^37^. Additionally, the skeleton density of *P. cylindrica* did not show significant differences among sites (Fig. S2), again contradicting previous studies that showed lower skeleton densities of corals at a CO_2_ vent^38^ and naturally acidified sites^39^. The net photosynthesis (Pn) rate of *P. cylindrica* transplanted to site N5 had a significantly higher value (*p* = 0.04) regardless of their origin (Fig. 5b, Table S8), which may be related to the slightly higher nutrient concentrations at N5 site (Table S7). Respiration (R) rates showed interactive effects among transplanted site and origin site, and the R rate of both M1 and N5 corals was significantly lower when transplanted to their origin site (Fig. 5c, Table S8). As a result, there were also interactive effects among the transplanted site and origin site with regards to gross photosynthesis (Pg):R, with higher values when transplanted to their original site (Fig. 5d, Table S8), indicating higher energy acquirement of corals at their own origin site. Interestingly, the corals *Acropora pulchra*, *Porites lutea* and *Coelastrea aspera* in a semi-enclosed lagoon of New Caledonia with low pH, high temperature, low oxygen conditions but high coral coverage, were found to exhibit lower calcification, higher respiration (R) and lower Pg:R compared to corals outside of the lagoon^25^. Acclimatization of corals at the New Caledonia lagoon was suggested to be caused by high respiration through potentially high heterotrophy of corals within the lagoon, which has high organic carbon sedimentation^25^. A comparatively high heterotrophy of the corals in Nikko Bay is also suggested as zooplankton abundance (particularly copepod abundance) was observed to be higher at site N5 compared to reference site M1 (Fig. 6), and this may partially alleviate the effects of high temperature and high CO_2_ by enhancing their energy availability^40,41^. However, taking into account that only the calcification rate of *P. cylindrica* at site M1 decreased when transplanted to site N5, potential epigenetic or genetic adaptation to the environmental conditions found within the bay appears to have occurred for Nikko Bay corals. This is also indicated by other findings that showed *Pocillopora acuta* within Nikko Bay had higher calcification rates when transplanted to their original site than out of the bay, while *P. acuta* from out of the bay were not able to survive when transplanted within the bay^42^.

*P. cylindrica* from N5 was also found to host two types of *Cladocopium* subclade C1^43^ (former *Symbiodinium* ‘Clade C’), as well as *Durusdinium*^43^ (former *Symbiodinium* ‘Clade D’), which are known to be tolerant to high temperatures^44^, while *P. cylindrica* from the other sites only hosted *Cladocopium* subclade C1 (Fig. S3). These differences in Symbiodiniaceae, particularly at the most sheltered Nikko Bay site, may be another adaptation mechanism of corals to the environment found within Nikko Bay. However, molecular studies evaluating the potential genetic differentiation of those host corals within the bay are first needed before implying the occurrence of local adaptation. Thus, for further understanding, molecular studies evaluating the potential genetic differentiation of these host corals within the bay are essential in evaluating of the possibility of local adaptation.

From the present study, coral community structure was found to change according to the seawater environmental conditions within the bay, and corals living within the bay such as *P. cylindrica* could maintain their fitness in the warmed and acidified conditions found within the bay. However, interpretation of these results as related to future climate change should be taken carefully, as several other environmental factors including Chl-*a*, DO, inorganic nutrient concentrations, and light intensities also varied among sites. Additionally, corals within this bay have been suggested to have been continuously exposed to the unique environment within Nikko Bay for at least the past 150–500 years^12^, while climate change is predicted to continue for the next few decades to centuries. Nevertheless, these results give important insights about the potential acclimatization and adaptation capacities of corals to different environmental conditions, even at small spatial scales, on coral reefs.

## Methods

### Water quality measurements

The carbonate chemistry including total alkalinity (TA), dissolved inorganic carbon (DIC), aragonite saturation (Ω_arag_), and water quality parameters including chlorophyll-*a* (Chl-*a*), turbidity (FTU), dissolved oxygen (DO) and inorganic nutrients (DIN, DIP) were measured once each during daytime and nighttime from 40 sites around Nikko Bay (Supplementary Fig. S1, Tables S1, S2).

Water quality measurements in Nikko Bay were conducted during daytime before sunset (15:00–18:00) and during nighttime around sunrise (5:00–8:00) between 17 to 19 November, 2014 at 40 sites (Fig. 1). Water samples were taken before sunset and after sunrise because of the following assumptions. Net primary production and calcification continue until compensation light intensity reaches near sunset, and therefore DIC and TA reach their minimum values around sunset in a closed system. On the other hand, respiration and dissolution continue until sunrise, so DIC and TA are at their maximum around sunrise in a closed system. Because of the highly closed nature of Nikko Bay, it is safe to assume that the range of diel changes in DIC and TA can be captured by collecting water at two time points, around sunset and around sunrise. As well, the middle value of these two time points can give the average value of the system. Seawater temperature, salinity, depth, Chl-*a* (fluorescent chloride sensor), dissolved oxygen (DO, phosphoresce based oxygen sensor) and turbidity (FTU, backscattering sensor, formazin reference) were measured by vertical casting (1 s logging interval) using a multi-parameter sensor (AAQ-Rinko, JFE Advantech). Salinity data was calibrated by a salinometer (PORTASAL 8410A, Guildline Instruments), and Chl-*a* (3 replicates) by a fluorometer (Trilogy, Turner Designs). Surface water samples for TA, DIC (2 replicates) and nutrient (DIN, DIP) measurements (4 replicates) were collected from the same sites. TA and DIC were measured within 24 h after seawater collection using an auto burette titrator (ATT-05, Kimoto Electronic Co. Ltd.), which has an accuracy of ± 2 µmol Kg^−1^ for TA and ± 3 µmol Kg^−1^ for DIC and standardized by certified reference materials obtained from A. Dickson Laboratory (Scripps Institution of Oceanography, Batch 139). When samples were not measurable within 24 h, they were poisoned with saturated mercury chloride and kept within air-tight glass vial bottles, and these samples were measured within a few days after collection. Nutrient samples were stored in a freezer (-20 °C) until measurement by nutrient autoanalyzer (AACSIII, BRAN + LUEBEE). Carbonate chemistry was calculated from measured TA, DIC and surface salinity and temperature measured at 30 cm below the seawater surface by a casting sensor using CO2SYS^45^ with the constants of Mehrbach et al.^46^ as refit by Dickson and Millero^47^, and aragonite solubility of Mucci^48^. The accuracy of calculated *p*CO_2_ is estimated as approximately 2–3% of the measurement range of Nikko Bay (see Anderson et al.^49^ for calculation of propagation error).

Mean net calcification and primary production in Nikko Bay were calculated from the differences between the average of inside Nikko Bay TA and DIC (n = 74 data each, Table S2) and offshore end-members, the average depth of the bay (d = 18 m), and the mean water residence time within the bay (τ = 71 days^12^), by simply assuming these TA and DIC differences were accumulated in the bay during this residence time; mean net calcification rate (Gn) was calculated from the TA differences, τ, d, and seawater density (ρ), and primary productivity (Pn) rate was calculated from DIC and TA differences, τ, d, and ρ (see Kayanne et al.^30^ and Watanabe et al.^50^). For the calculation TA and DIC were normalized at S = 33.02 which was the mean salinity of Nikko Bay.

In addition to seawater multi-station measurements across the entire bay, several measurements were conducted during the subsequent benthic survey and coral transplantation survey. Seawater qualities (3 m depth) for the seven sites where benthic surveys were conducted were measured using the AAQ-Rinko in the same manner as above. Additionally, seawater samples were taken at 3 m depths using Van Dorn samplers to measure TA, DIC, and nutrients. During coral transplantation, bottom seawater was sampled using Van Dorn samplers on October 17, 2015 for measurement of Chl-*a* (3 replicates), nutrients (4 replicates), and suspended solids (3 replicates). Seawater carbonate chemistry (TA and DIC) was measured by collecting two replicate water samples three times (17 Oct, 28 Oct and 5 Nov, 2015) during transplantation. pH and temperature loggers (SPS-14, Kimoto Electrode, Japan) were also deployed at the three transplantation sites for ca. 5 days. The pH data for the loggers were calibrated using TRIS-AMP buffers and are reported in the total scale.

### Benthic community survey

Benthic coverage including coral community cover (at the genus level) was determined by taking 0.5 × 0.5 m photo-quadrats every meter along five 50 m transects at 3 m depth. A total of 50 photographs were taken per transect and per depth at each site; representing an area of 12.5 m^2^ of benthos per depth and site. We measured coral community composition and water quality of one site outside of Nikko Bay (N1) and six sites within Nikko Bay (N2-N7) at similar depths (~ 5 m) showing different Ω_arag_ conditions (1.39–3.45) for the analyses.

Sites N1 to N5 were surveyed in November 2015 while sites N6 and N7 were surveyed in August 2014. No major disturbances within Nikko Bay (e.g. bleaching events) were observed between the two benthic survey dates. Benthic photographs were analyzed using CPCe software^51^. The benthic substrate directly below five random points per photograph was classified into benthic categories as described in several previous studies (e.g. Houk and van Woesik^52^, Golbuu et al.^14^, Gouezo et al.^17^, Houk et al.^15^). The benthic categories included live corals (to the genus level), fleshy macroalgae (identified to the genus level), turf algae, other invertebrates (e.g. sponges, ascidians, gorgonians), crustose coralline algae (CCA), and non-living substrates (e.g. bare rock, rubble, sand). The percentage cover of benthic categories at each site and depth was the result of the average of percentage cover among the five transects.

### Transplantation experiment

Two nubbins of 5 cm length were collected by cutting pliers from each of 12 *Porites cylindrica* colonies in September 2015 from Nikko Bay (sites N5) and a reference site at Malakal Bay (site M1) at 2–3 m depths, respectively. The M1 site was selected as reference site because the coral community structure was similar to Nikko Bay and dominated by *P. cylindrica*, while seawater carbonate chemistry and seawater temperatures were close to the conditions found outside the bay such as at site N1. Sampled corals were put within a container with seawater bubbled with an air-pump, and transported by boat after sampling to the Palau International Coral Reef Center, arriving within 1 h (PICRC, Fig. 1b, c). After bringing all samples to the center, coral nubbins were each glued individually onto the top of plastic bolts using epoxy glue, and transplanted back to the same site from which they were collected. After one month, all nubbins were recovered and buoyant wet weights were measured with an electronic balance (0.1 mg precision HR-200, A&D, Japan) twice for each nubbin. Thereafter, coral nubbins were reciprocally transplanted to the two sites, with each one nubbin of 12 colonies collected from the 2 sites transplanted to each 2 sites (n = 24 per site). During transplantation, both seawater temperatures and light intensities were logged at 10 min intervals using temperature (CO-UA-002, HOBO, Onset Corp.) and light sensor loggers (DEFI-L, JFE Advantech, cleaned every weak), respectively.

Eighteen days after transplantation, all nubbins were recovered and the buoyant wet weights of all nubbins were measured. Skeletal dry weights were calculated using aragonite density (2.94 g cm^−3^) according to Davis^53^, and calcification rates were calculated by the change of skeletal dry weights during transplantation and normalized by the surface areas measured using the aluminum foil technique^54^. Additionally, photosynthesis and respiration rates were measured for 9 out of 12 nubbins in each condition. Coral nubbins (36 nubbins in total) were first cleaned to remove any attached algae on the coral surface, and each nubbin was then placed individually in an airtight glass container (volume = 400 ml) filled with seawater collected from the same site where the corals were transplanted, placed under LED light (250 μmol photon m^−2^ s^−1^) and photosynthesis was measured during daytime (11:00–14:00). One extra incubation container without a nubbin was also added as a control. Seawater within the container was continuously stirred with a magnetic stirrer (450 rpm) during incubation and oxygen concentrations were measured at 0, 20 and 40 min using an oxygen sensor (Fibox3, PreSens) calibrated with 100% saturated and 0% oxygen water prepared by Na_2_SO_3_. The same procedure was also conducted at night (20:00–22:00) under dark conditions to measure the respiration rate. Net photosynthesis (Pn) and respiration (R) rates were calculated by the following equation, where V is volume (ml), T is time in hours, and SA is the surface area of the coral (cm^−2^). Gross photosynthesis (Pg) was calculated by Pn minus R.

$$ {\text{Pn}}\;{\text{or}}\;{\text{R}}\;\left( {\mu {\text{mol}}\;{\text{O}}_{2} \;{\text{cm}}^{ - 2} \;{\text{h}}^{ - 1} } \right) = \Delta \left[ {{\text{O}}_{2} } \right]*\left( {{\text{V}}_{{{\text{chamber}}}} - {\text{V}}_{{{\text{coral}}}} } \right)/{\text{T}}*{\text{SA}} $$

Seawater volume versus coral nubbin size was care to minimize the change in carbonate chemistry of the seawater within the container during the incubation due to the photosynthesis and respiration by the corals, and the seawater pH change was kept to be smaller than 0.08, which gives in average *p*CO_2_ change of 200 μmol.

### Zooplankton

Zooplankton were sampled at night after sunset for three days (11, 12 and 13 March 2016) from three sites (N5, N7, and M1) using a 100 m Nansen plankton net (30 cm diameter). Horizontal tows were conducted 5 times at 1–3 m depth per site, and the filtered volume was recorded with a flowmeter. Zooplankton samples were split and one-half of the sample was preserved in 5% borated-buffered formalin. Individual numbers of all zooplankton and copepods of the formalin fixed samples were counted under a microscope.

### Statistical methods

Differences in seawater qualities inside and outside Nikko Bay were analyzed using Wilcoxon signed-rank test. Redundancy analysis (RDA) was conducted as a constrained ordination technique to relate the coral communities to seawater environmental variables. Input for the RDA consisted of coral coverage data that were first transformed using the decostand function in the R package vegan^55^. In the present case, the Hellinger distance was used. Generalized linear models (GLMs, family = quasipoisson) were used to evaluate the relation between Ω_arag_ and coral and other benthic coverage. The calcification rates of the transplantation experiment were evaluated with a Generalized Linear Mixed Effects model (family = Gamma) with origin site and transplanted site and its interaction as fixed effects and colony as random effect. Net photosynthesis, respiration (log transformed), and Pg:R (log transformed) of the transplantation experiment were evaluated with a linear mixed-effect model (REML) with origin site and transplanted site and its interaction as fixed effects and colony as random effect. Tukey’s HSD test was conducted when there was a significant interaction. Differences of zooplankton and copepod abundances among sites were tested with student t-test. All statistical analyses were conducted using R (version 3.6.3)^56^.

## Supplementary Information

Supplementary Information 1.

Supplementary Information 2.
