# Author Correction: Potential local adaptation of corals at acidified and warmed Nikko Bay, Palau

**DOI:** 10.1038/s41598-021-96624-w

**Published:** 2021-08-24

**Authors:** Haruko Kurihara, Atsushi Watanabe, Asami Tsugi, Izumi Mimura, Chuki Hongo, Takashi Kawai, James Davis Reimer, Katsunori Kimoto, Marine Gouezo, Yimnang Golbuu

**Affiliations:** 1 Department of Chemistry, Biology, and Marine Science, Faculty of Science, University of the Ryukyus, 1 Senbaru, Nishihara, Okinawa 903-0213 Japan; 2 Department of Transdisciplinary Science and Engineering, School of Environment and Society, Tokyo Institute of Technology, 2-12-1 W8-13, Meguro, Tokyo 152-8550 Japan; 3 The Ocean Policy Research Institute, The Sasakawa Peace Foundation, 1-15-16 Toranomon, Tokyo, Minato 105-8524 Japan; 4 Research Institute for Global Change, Japan Agency for Marine-Earth Science and Technology (JAMSTEC), 2-15, Natsushima-cho, Yokosuka, Kanagawa 237-0061 Japan; 5 Palau International Coral Reef Center, 1 M-Dock Road, PO Box 7086, Koror, 96940 Republic of Palau

Correction to: *Scientific Reports* 10.1038/s41598-021-90614-8, published online 27 May 2021

The original version of this Article contained an error in Figure 1a, where the location of Nikko Bay as indicated on the map was incorrect. The original Figure 1 and accompanying legend appear below.

**Figure 1:**
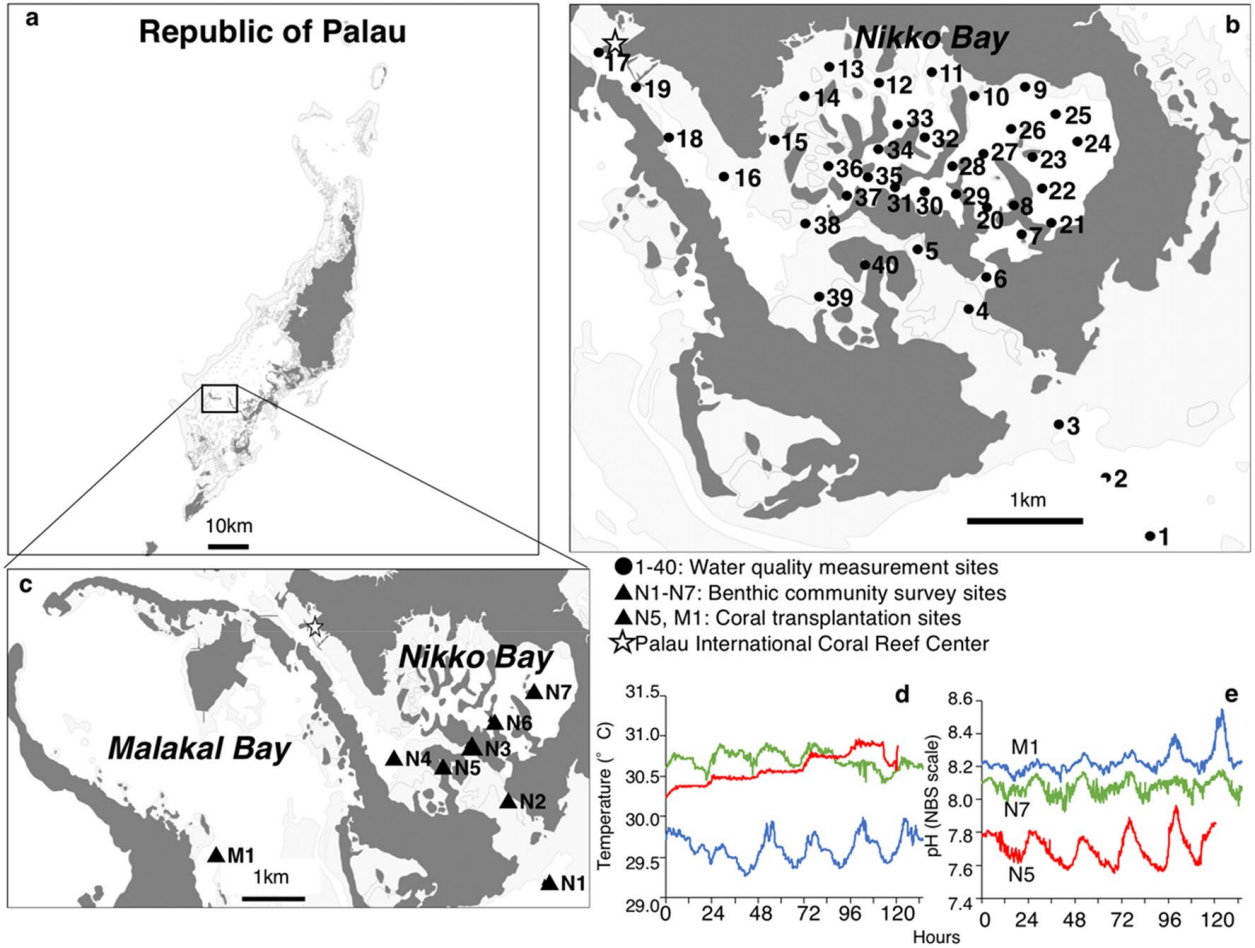
Map showing study sites and seawater temperature and pH at 3 sites (M1, N7, N5). (**a**) Map of the Republic of Palau. (**b**) The 40 locations where seawater quality was measured around Nikko Bay. (**c**) The seven sites (N1-N7) where benthic communities were surveyed and the reference site at Malakal Bay (M1) where the coral *Porites cylindrica* experiment was conducted. The coral *P. cylindrica* was sampled from sites M1 and N5 for reciprocal transplantation experiment. (**d**) Diurnal seawater temperature and (**e**) pH (total scale) measured at Malakal Bay (M1) and two sites at Nikko Bay (N7 and N5). The figure was created using QGIS 3.8.1 (https://www.qgis.org).

The original Article has been corrected.

