# Supplementary material for: Potential local adaptation of corals at acidified and warmed Nikko Bay, Palau
Source: Sci Rep. 2021 May 27;11:11192. doi: 10.1038/s41598-021-90614-8 (PMC8159998; doi:10.1038/s41598-021-90614-8)
Supplement: Supplementary file 1 — Supplementary Information 1. [file 41598_2021_90614_MOESM1_ESM.docx]

**SUPPLEMENTARY METHODS**

**Potential local adaptation of corals at acidified and warmed Nikko Bay, Palau**

Haruko Kurihara^1^*, Atsushi Watanabe^2,3^, Asami Tsugi^1^, Izumi Mimura^1^, Chuki Hongo^1^, Takashi Kawai ^1^, James Davis Reimer^1^, Katsunori Kimoto^4^, Marine Gouezo^5^, Yimnang Golbuu^5^

1. Department of Chemistry, Biology, and Marine Science, Faculty of Science, University of the Ryukyus, 1 Senbaru, Nishihara, Okinawa 903-0213, Japan

2. Department of Transdisciplinary Science and Engineering, School of Environment and Society, Tokyo Institute of Technology, 2-12-1 W8-13, Meguro, Tokyo 152-8550, Japan

3. The Ocean Policy Research Institute, The Sasakawa Peace Foundation, 1-15-16 Toranomon, Minato, Tokyo 105-8524, Japan

4. Research Institute for Global Change, Japan Agency for Marine-Earth Science and Technology (JAMSTEC), 2-15, Natsushima-cho, Yokosuka, 237-0061, Japan

5. Palau International Coral Reef Center, 1 M-Dock Road, PO Box 7086, Koror, PW 96940, Republic of Palau

*Corresponding author: harukoku@sci.u-ryukyu.ac.jp, harukoku@e-mail.jp

**Skeleton density.**

Skeleton density analysis was performed by following methods: First, volume of skeleton of coral was measured by the Micro-focus X-ray Computed Tomography (ScanXmateD160TSS650, Comscantechno Co.Ltd., Japan) equipped in Japan Agency for Marine-Earth Science and Technology (JAMSTEC). High resolution settings (X-ray focus diameter was 0.8 µm; X-ray tube voltage was 90 KeV and tube current was 35 µA; detector array size of 1464 x 1152 pixels; 1800 projections in 360 rotations) were applied for 3D quantitative volumetry of individual corals. Geometric resolution of isotropic voxel was 35 µm. We used ConeCTexpress (Comscantecno Co. Ltd., Japan) for correction and reconstruction tomography data and general principle of Feldkamp cone beam reconstruction was followed to reconstruct image cross sections based on filtered back projections. Second, weight of coral was measured by electric precision balance (AT201, Mettler Toledo International Inc., USA) and 3-times average value was used. Finally, skeleton density of coral was calculated by dividing the weight of coral by its volume.

**Identification of Symbiodiniaceae**

The amplification of Symbiodiniaceae followed Wee et al. (2019). In brief, genomic DNA was extracted from specimens using a DNeasy Blood and Tissue extraction kit following the manufacturer’s instructions (Qiagen, Tokyo). Two DNA marker regions were amplified via polymerase chain reaction (PCR): the internal transcribed spacer 2 region (ITS2) of nuclear ribosomal DNA and the non-coding region of the plastid minicircle (psbA^ncr^). The ITS2 sequences from this study were utilized to place symbiont types within the well-established ITS2 phylogenetic framework (LaJeunesse and Thornhill 2011). The psbA^ncr^ sequences were utilized to examine differences at a finer phylogenetic resolution (LaJeunesse and Thornhill 2011; Reimer et al. 2017; Noda et al. 2017).

The ITS2 region was amplified using previously published primers (White et al. 1990; Rowan and Powers 1992; Hunter et al. 1997), as was psbA^ncr^ (LaJeunesse and Thornhill 2011). PCR thermocycle conditions were modified slightly from Noda et al. (2017): ITS2: 95.0ºC for 5 min; 35 cycles of 94.0ºC for 30 s, 51.0ºC for 45 s, and 72.0ºC for 2 min; with a final extension at 72.0ºC for 10 min; and for psbA^ncr^: 95.0ºC for 5 min; 40 cycles of 94.0ºC for 10 s, 55.0ºC for 30 s, and 72.0ºC for 2 min; with a final extension at 72.0ºC for 10 min. Products were sequenced in both directions by Fasmac (Kanagawa, Japan).

The nucleotide sequences of ITS2 and psbA^ncr^ acquired were edited and aligned separately within Geneious v9.1.8 (Biomatters Ltd.). Each alignment was inspected manually, and primer regions and uneven tail ends were excluded. The reverse reads of psbA^ncr^ were used for examining genotypes in this study, as in Noda et al. (2017). Sequences were then genotyped, and identified to *Cladocopium* subclade level (ITS2) or to different *Cladocopium* lineages (psbA^ncr^) based on generated alignments. No further phylogenetic analyses were conducted on these sequences.

**References**

Hunter, C.L. The utility of ITS sequences in assessing relationships among zooxanthellae and corals. *Proc. 8th Int. Coral Reef Symp.* (22): 1599–1602 (1997)

LaJeunesse, T.C. & Thornhill, D.J. Improved resolution of reef coral endosymbiont (*Symbiodinium*) species diversity, ecology, and evolution through psbA non-coding region genotyping. *PLoS One* **6**(12), e29013 (2011).

Noda, H., Parkinson, J.E., Yang, S.Y. & Reimer, J.D. A preliminary survey of zoantharian endosymbionts shows high genetic variation over small geographic scales on Okinawa-jima Island, Japan. *PeerJ* **5**, e3740 (2017).

Reimer, J.D., Herrera, M., Gatins, R., Roberts, M.B., Parkinson, J.E. & Berumen, M.L. Latitudinal variation in the symbiotic dinoflagellate *Symbiodinium* of the common reef zoantharian *Palythoa tuberculosa* on the Saudi Arabian coast of the Red Sea. *J. Biogeogr.* **44**(3), 661–673 (2017).

Rowan, R. & Powers, D. Molecular genetic identification of symbiotic dinoflagellates (zooxanthellae). *Mar. Ecol. Prog. Ser.* **71**(1), 65–73 (1991).

White, T.J., Bruns, T., Lee, S.J.W.T. & Taylor, J.L. Amplification and direct sequencing of fungal ribosomal RNA genes for phylogenetics. *PCR protocols: a guide to methods and applications* **18**(1), 315–322 (1990).
