# Supplementary material for: Potential local adaptation of corals at acidified and warmed Nikko Bay, Palau
Source: Sci Rep. 2021 May 27;11:11192. doi: 10.1038/s41598-021-90614-8 (PMC8159998; doi:10.1038/s41598-021-90614-8)
Supplement: Supplementary file 2 — Supplementary Information 2. [file 41598_2021_90614_MOESM2_ESM.docx]

**SUPPLEMETARY MATERIAL**

**Potential local adaptation of corals at acidified and warmed Nikko Bay, Palau**

Haruko Kurihara^1^*, Atsushi Watanabe^2,3^, Asami Tsugi^1^, Izumi Mimura^1^, Chuki Hongo^1^, Takashi Kawai ^1^, James Davis Reimer^1^, Katsunori Kimoto^4^, Marine Gouezo^5^, Yimnang Golbuu^5^

1. Department of Chemistry, Biology, and Marine Science, Faculty of Science, University of the Ryukyus, 1 Senbaru, Nishihara, Okinawa 903-0213, Japan

2. Department of Transdisciplinary Science and Engineering, School of Environment and Society, Tokyo Institute of Technology, 2-12-1 W8-13, Meguro, Tokyo 152-8550, Japan

3. The Ocean Policy Research Institute, The Sasakawa Peace Foundation, 1-15-16 Toranomon, Minato, Tokyo 105-8524, Japan

4. Research Institute for Global Change, Japan Agency for Marine-Earth Science and Technology (JAMSTEC), 2-15, Natsushima-cho, Yokosuka, 237-0061, Japan

5. Palau International Coral Reef Center, 1 M-Dock Road, PO Box 7086, Koror, PW 96940, Republic of Palau

*Corresponding author: harukoku@sci.u-ryukyu.ac.jp, harukoku@e-mail.jp

**
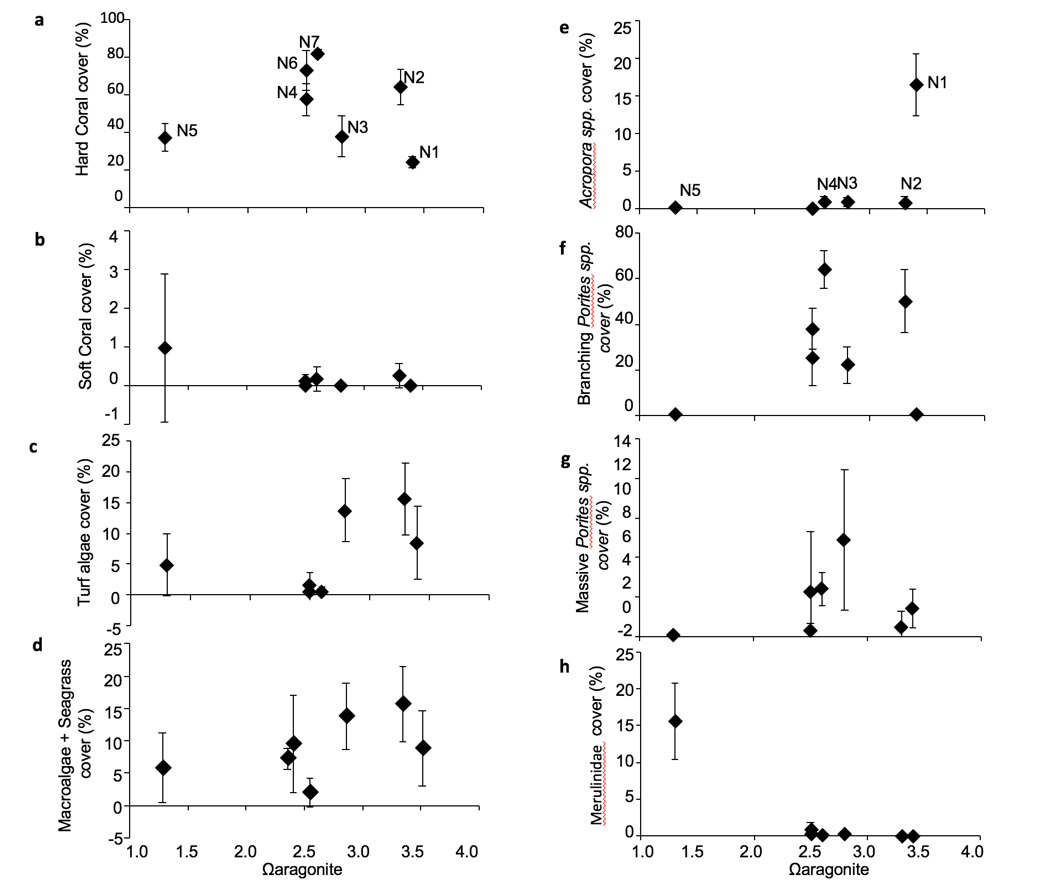
**

**Figure S1. Benthic cover and hard coral richness change across the aragonite saturation (Ω_arag_) gradient. a.** Hard coral cover, **b.** Soft coral cover, **c.** Turf algae cover, **d.** Macroalgae plus seagrass cover, **e.** *Acropora* spp. cover, **f.** Massive *Porites* spp. cover, **g**. Branching *Porites* spp. cover, and **h**. Merulinidae cover. Mean (n = 5 transect) ± SD.

**
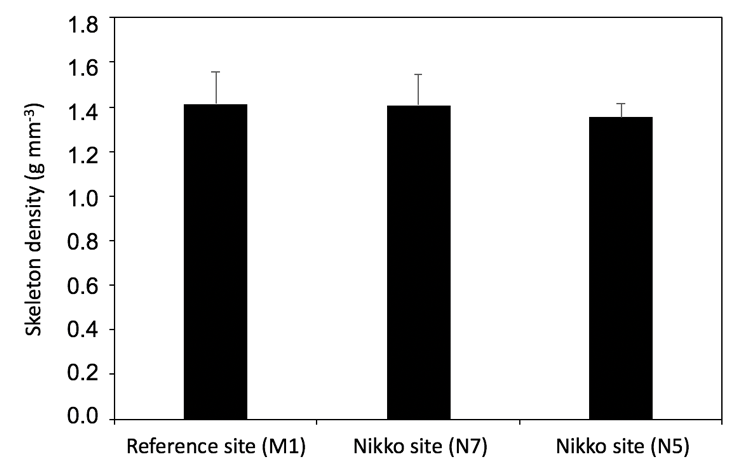
**

**Figure S2**. **Skeletal densities of the coral *Porites cylindrica* originating from reference site (M1) and Nikko Bay site (N7 and N5).** There was no significant difference in skeleton density of *P. cylindrica* among sites that showed different seawater *p*CO_2_/Ω_arag_ conditions.

**
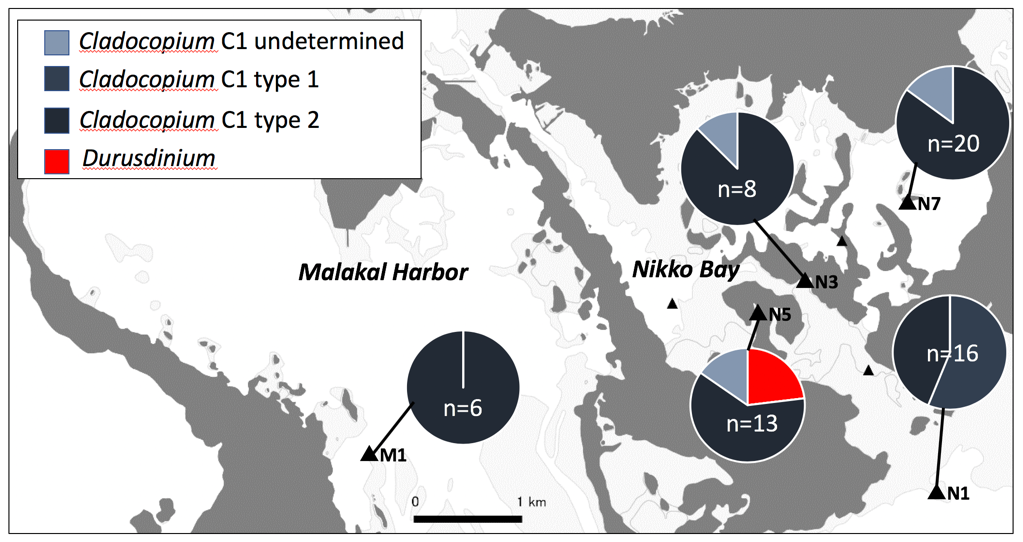
**

**Figure S3. Symbiodiniaceae types of *Porites cylindrica* from different sites within and outside of Nikko Bay.** While *P. cylindrica* mainly hosted *Cladocopium* subclade C1, *P. cylindrica* corals from site N5 hosted both *Cladocopium* subclade C1 and *Durusdinium.* The figure is created using QGIS 3.8.1 (https://www.qgis.org).

**Table S1.** Environmental conditions for surface water measured at 40 sites (Site 1-3: outside Nikko Bay, Site 4-40: within Nikko Bay) during daytime and nighttime. Daytime measurements were conducted before sunset (15:00-18:00) and nighttime measurements were conducted around sunrise (5:00-8:00). Temperature, salinity, chlorophyll-*a* (Chl-*a*), turbidity and dissolved oxygen (DO) data are the data measured using the multi-parameter sensor (AAQ-Rinko) at 30 cm below the surface. Nutrient data are the average of 4 samples collected at surface using bucket as described in methods. Average, maximum and minimum values of the data measured within Nikko Bay (Site4-40) are also described at the bottom of the table.

**
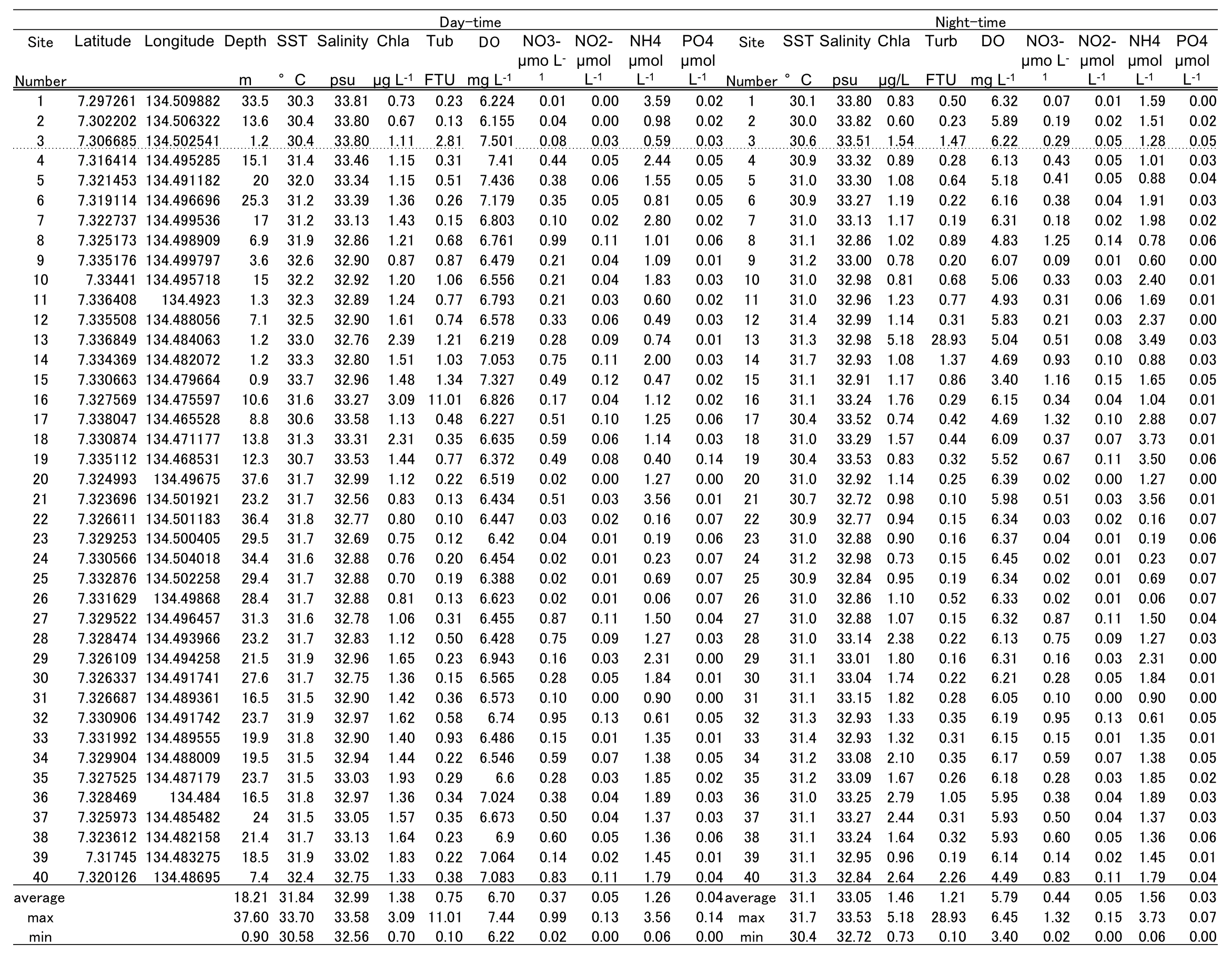
**

**Table S2.** Carbonate chemistry measured at the same 40 sites during daytime and nighttime described at Table S1. Total alkalinity (TA) and dissolved inorganic carbon (DIC) data are the average of 2 samples collected at surface as described in the methods. Seawater pH (total scale), pCO_2_, HCO_3_^-^, CO_3_^2-^ and Ω_arag_ were calculated by TA, DIC, temperature, and salinity using the CO2SYS program of Lewis and Wallace (1998), with dissociation constants K1 and K2 from Mehrbach et al. (1973) and the aragonite solubility of Mucci (1983). Average, maximum and minimum values of the data measured within Nikko Bay (sites 4-40) are also described at the bottom of the table.

**
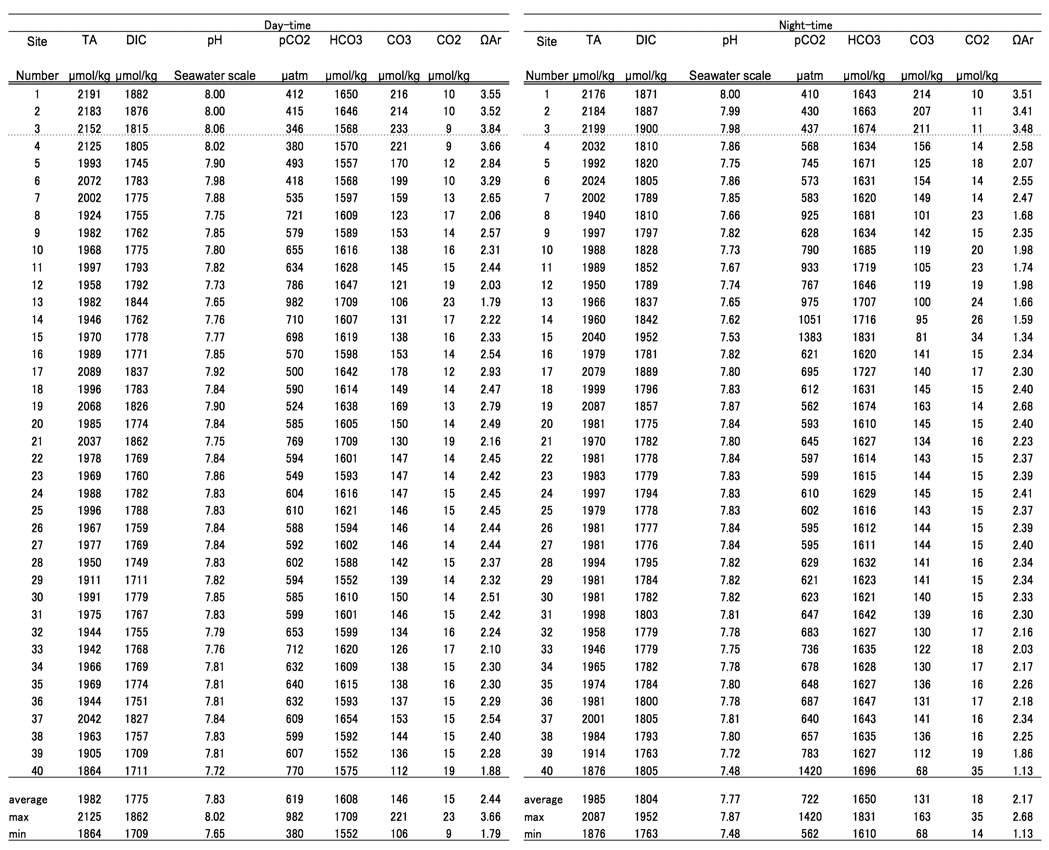
**

**Table S3.** Environmental conditions at 3 m depth water at 7 sites (N1: outside Nikko Bay and N2-N7: within Nikko Bay). Temperature, salinity, Chl-*a*, turbidity and DO data were measured by multi-parameter sensor. Nutrient data are the average of 4 samples and total alkalinity (TA) and dissolved inorganic carbon (DIC) data are the average of 2-3 samples. Water samples were collected using Van Dorn-type water sampler. Seawater pH (total scale), pCO_2_, and Ω_arag_ were calculated from TA, DIC, temperature, and salinity using the CO2SYS program of Lewis and Wallace (1998), with dissociation constants K1 and K2 from Mehrbach et al. (1973) and the aragonite solubility of Mucci (1983).

| Site | Latitude | Longitude | SST | Salinity | Chl-*a* | Turb | DO | NO_3_^-^ | NO_2_^-^ | NH_4_ | PO_4_^3-^ |
| --- | --- | --- | --- | --- | --- | --- | --- | --- | --- | --- | --- |
| Name | N | E | °C | psu | μg/L | FTU | mg/l | μmol/L | μmol/L | μmol/L | μmol/L |
| N1 | 7.30428 | 134.50246 | 29.29 | 34.15 | 0.55 | 0.42 | 6.09 | 0.84 | 0.04 | 0.28 | 0.07 |
| N2 | 7.31488 | 134.49670 | 29.37 | 34.09 | 0.48 | 0.45 | 6.73 | 0.30 | 0.03 | 0.52 | 0.06 |
| N3 | 7.32248 | 134.49096 | 30.05 | 33.82 | 0.94 | 0.44 | 6.60 | 1.01 | 0.04 | 0.46 | 0.08 |
| N4 | 7.32061 | 134.47974 | 30.33 | 33.76 | 1.40 | 0.41 | 5.90 | 0.29 | 0.00 | 0.71 | 0.06 |
| N5 | 7.32010 | 134.48711 | 30.49 | 33.45 | 1.68 | 0.83 | 4.52 | 0.34 | 0.10 | 0.63 | 0.05 |
| N6 | 7.32602 | 134.49439 | 30.41 | 32.87 | 1.57 | 0.26 | 6.12 | 0.73 | 0.11 | 1.10 | 0.09 |
| N7 | 7.32602 | 134.49439 | 30.31 | 33.22 | 0.96 | 0.38 | 6.48 | 0.55 | 0.06 | 0.83 | 0.07 |

| Site | TA | DIC | pH | *p*CO_2_ | ΩAr |
| --- | --- | --- | --- | --- | --- |
| Name | μmol/kg | μmol/kg | Total scale | μatm |  |
| N1 | 2174 | 1867 | 8.03 | 395 | 3.51 |
| N2 | 2154 | 1861 | 8.00 | 418 | 3.35 |
| N3 | 2132 | 1888 | 7.91 | 537 | 2.86 |
| N4 | 2076 | 1861 | 7.86 | 604 | 2.55 |
| N5 | 1955 | 1871 | 7.54 | 1305 | 1.28 |
| N6 | 2019 | 1817 | 7.85 | 602 | 2.41 |
| N7 | 2000 | 1802 | 7.84 | 612 | 2.36 |

**Table S4.** Mean and standard deviation (S.D.) of hard corals, soft corals, macroalgae + seagrass, turf algae and crustose coralline algae (CCA) cover (%), coral richness (number of genera per transect), and cover of each hard coral genus (%) for the 5 transects measured at 7 sites (N1: outside Nikko Bay and N2-N7: within Nikko Bay). Merulinidae are the sum of all genera of the family Merulinidae excluding *Dipsastraea* spp*., Favites* spp*., Goniastrea* spp*., Montastrea* spp*.* and *Echinopora* spp*. Porites* spp*.* was divided into massive *Porites* spp., branching *Porites* mainly consisting of *Porites cylindrica,* and *Porites rus.*


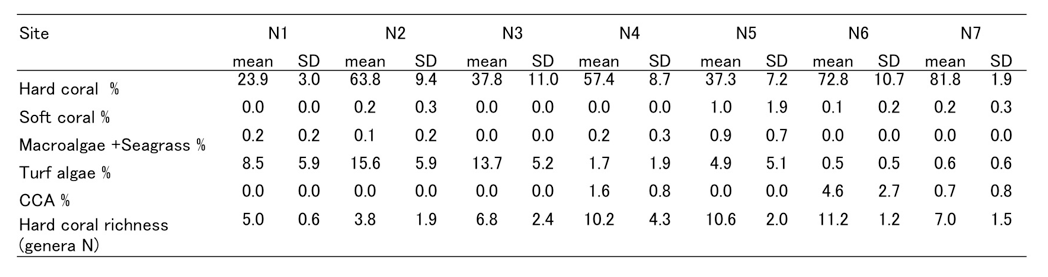


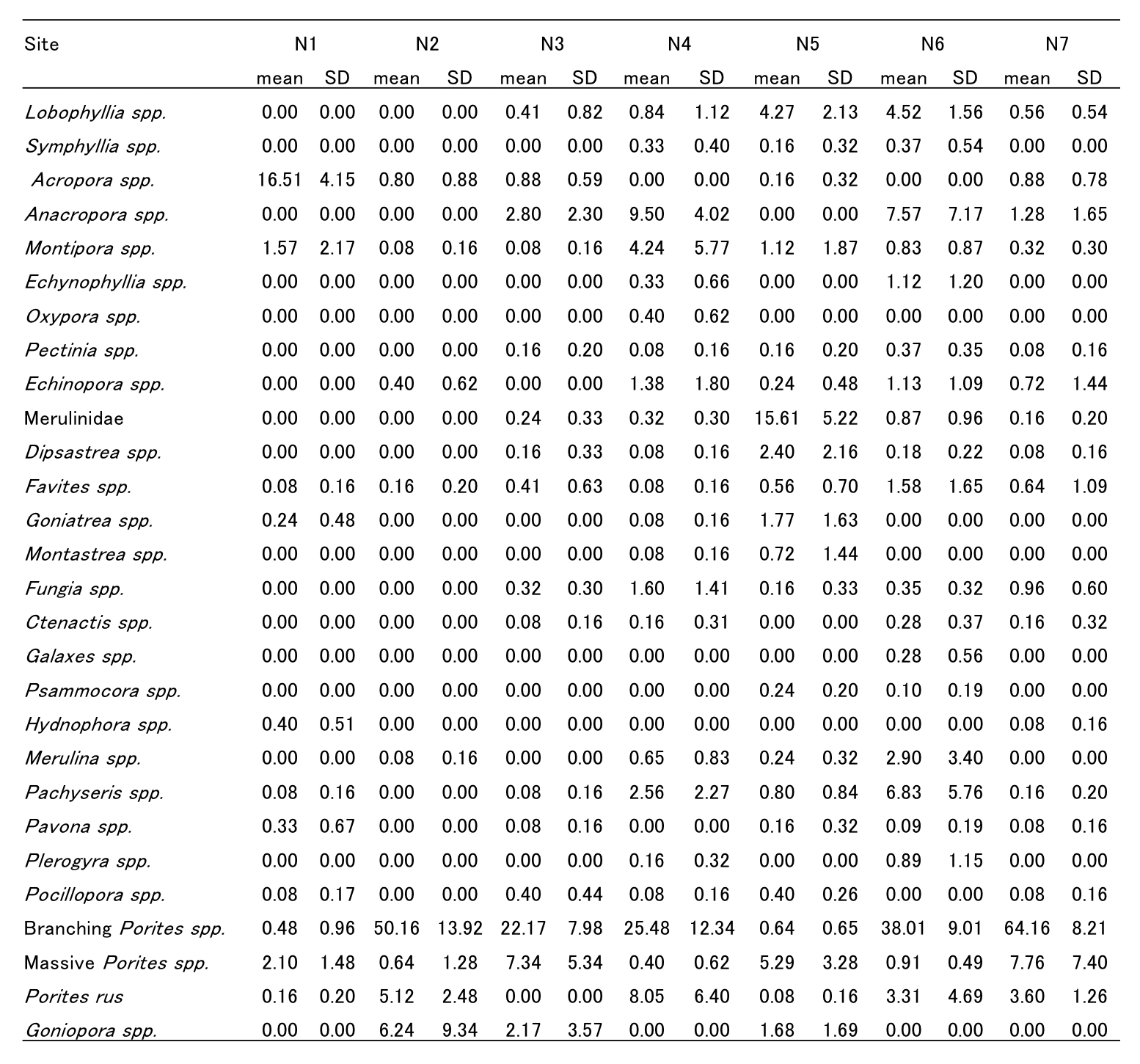


**Table S5.** GLM analysis of benthic cover (%) and hard coral richness (number of genera) along the Ω_arag_ gradient at 7 sites (N1: outside Nikko Bay and N2-N7: within Nikko Bay).

|  | **Estimate** | **SE** | **t-value** | ***p*-value** |
| --- | --- | --- | --- | --- |
| Hard coral (%) | -0.003 | 0.1 | -0.03 | 0.97 |
| Soft coral (%) | -1.49 | 0.59 | -2.52 | **0.01** |
| Macroalgae+Seagrass (%) | 0.41 | 0.22 | 1.89 | 0.06 |
| Turf (%) | 0.77 | 0.36 | 2.12 | **0.04** |
| CCA(%) | -0.2 | 0.49 | -0.41 | **0.68** |
| Acropora spp. (%) | 0.77 | 0.36 | 2.12 | **0.04** |
| Massive *Porites spp.* (%) | 0.77 | 0.36 | 2.12 | **0.04** |
| Branching *Porites spp*. (%) | 0.34 | 0.24 | 1.43 | 0.16 |
| Faviidae (%) | 0.77 | 0.36 | 2.12 | **0.04** |

**Table S6.** Portion of the variation explained by each significant environmental variable by redundancy analysis (RDA).

| Variables | **%** | ***F*** | ***p*** |
| --- | --- | --- | --- |
| DO | 22.9 | 11.09 | <0.01 |
| Ω_arag_ | 19.9 | 12.47 | <0.01 |
| NO_3_^-^+NO_2_^-^ | 7.8 | 6.02 | <0.01 |
| Chl-*a* | 9.4 | 8.31 | <0.01 |
| *p*CO_2_ | 4.4 | 4.67 | <0.01 |
| Seawater temperature | 1.9 | 2.61 | <0.01 |

**Table S7.** Environmental conditions at the reference site (M1: Malakal Bay) and Nikko Bay site (N5) measured during the transplantation experiment of the coral *Porites cylindrica*. Temperature (SST) and light intensity is mean ± S.D. of the data measured every 10 minutes using temperature and light sensor logger during transplantation. Salinity and DO were measured using a multi-parameter sensor. Water samples were collected using Van Dorn-type water sampler on day 17 Oct for Chl-*a* (n =3), suspended solid (SS, n = 3), and nutrients (n = 4). SS was measured by filtering 10 L of seawater using pre-combusted and weighed GF/F glass filters. For TA (n = 6) and DIC (n = 6), 2 replicate water samples were collected using Van Dorn-type on day 17, 18 Oct and 5 Nov, 2015. Seawater pH (total scale), pCO_2_^-^ and Ω_arag_ were calculated by TA, DIC, temperature, and salinity using the CO2SYS program of Lewis and Wallace (1998), with disassociation constants K1 and K2 from Mehrbach et al. (1973) and the aragonite solubility of Mucci (1983).

| Site | Latitude | Longitude | SST | | | Salinity | | | Chl-*a* | | | SS | | | DO | | |
| --- | --- | --- | --- | --- | --- | --- | --- | --- | --- | --- | --- | --- | --- | --- | --- | --- | --- |
| Name | N | E | °C | | | psu | | | μg/L | | | mg/L | | | mg/L | | |
| M1 | 7.30766 | 134.4555 | 29.5 | ± | 0.2 | 33.7 | ± | 0 | 0.79 | ± | 0.00 | 0.59 | ± | 0.19 | 6.0 | ± | 0.7 |
| N5 | 7.32010 | 134.4871 | 30.6 | ± | 0.2 | 33.2 | ± | 0 | 2.22 | ± | 0.53 | 0.91 | ± | 0.10 | 3.8 | ± | 1.1 |

| Site | NO_3_- | | | NO_2_- | | | NH_4_ | | | PO_4_^3-^ | | |
| --- | --- | --- | --- | --- | --- | --- | --- | --- | --- | --- | --- | --- |
| Name | μmol/L | | | μmol/L | | | μmol/L | | | μmol/L | | |
| M1 | 0.60 | ± | 0.16 | 0.05 | ± | 0.00 | 3.04 | ± | 1.89 | 0.05 | ± | 0.00 |
| N5 | 0.43 | ± | 0.16 | 0.00 | ± | 0.00 | 1.64 | ± | 1.01 | 0.03 | ± | 0.00 |

| Site | TA | | | DIC | | | pH | | | *p*CO_2_ | | | ΩAr | | | light intensity |
| --- | --- | --- | --- | --- | --- | --- | --- | --- | --- | --- | --- | --- | --- | --- | --- | --- |
| Name | μmol/kg | | | μmol/kg | | | Total scale | | | μatm | | |  | | | μmol photon m^-2^s^-1^ |
| M1 | 2161 | ± | 12 | 1889 | ± | 7 | 8.0 | ± | 0.01 | 470 | ± | 9 | 3.2 | ± | 0.1 | 206 |
| N5 | 1898 | ± | 13 | 1809 | ± | 13 | 7.6 | ± | 0.03 | 1218 | ± | 113 | 1.3 | ± | 0.1 | 119 |

**Table S8.** Statistical parameters for calcification rate (G), net photosynthesis (nP), respiration (R) and gross photosynthesis ratio to respiration rate (gP : R) of corals collected at reference site (M1) and Nikko Bay site (N5) and reciprocally transplanted to M1 and N5 sites. Transplanted site and origin site were evaluated as fixed effects, and colony as random effect. Significant fixed effects (*p* <0.05) are shown in bold.

|  | df | *F* | *p* |
| --- | --- | --- | --- |
| **Calcification (G)** |  |  |  |
| Transplant site | 1 | 19.52 | **<0.0001** |
| Origin site | 1 | 0.51 | 0.75 |
| Transplant X Origin | 1 | 5.29 | **0.01** |
|  |  |  |  |
| **net Photosynthesis (nP)** |  |  |  |
| Transplant site | 1 | 4.62 | **0.04** |
| Origin site | 1 | 0.77 | 0.39 |
| Transplant X Origin | 1 | 0.13 | 0.71 |
|  |  |  |  |
| **Respiration (R)** |  |  |  |
| Transplant site | 1 | 1.24 | 0.28 |
| Origin site | 1 | 2.93 | 0.10 |
| Transplant X Origin | 1 | 34.8 | **<0.0001** |
|  |  |  |  |
| **gP/R** |  |  |  |
| Transplant site | 1 | 1.82 | 0.19 |
| Origin site | 1 | 2.24 | 0.15 |
| Transplant X Origin | 1 | 10.8 | **0.004** |

References

Lewis, E. & Wallace, D. CO2SYS: program developed for the CO_2_ system calculations. Carbon Dioxide Inf. Anal. Center. Oak Ridge National Laboratory, US Department of Energy, Oak Ridge, TN, USA (1998).

Mehrbach, C., Culberson, C. H., Hawley, J. E. & Pytkowicz, R. M. Measurement of the apparent dissociation constant of carbonic acid in seawater at atmospheric pressure. *Limnol. Oceanog*. **18**, 897-907 (1973).

Mucci, A. The solubility of calcite and aragonite in seawater at various salinities, temperatures, and one atmosphere total pressure. *Am. J. Sci.* **183**, 780-799 (1983).
